# Solid and Cystic Tumor (SCT) of the Pancreas in an Adult Man

**DOI:** 10.1155/1997/61689

**Published:** 1997

**Authors:** K. Ohiwa, M. Igarashi, N. Nagasue, M. Nagasaki, T. Harada

**Affiliations:** 1 Department of Surgery Muikaichi Hospital Muikaichi Japan; 2 Department of Surgery 2nd Unit Shimane Medical University Izumo Japan; 3 Department of Pathology 1st Unit Shimane Medical University Izumo Japan; 4 Department of Pathology 2nd Unit Shimane Medical University Izumo Japan

## Abstract

Solid and cystic tumor (SCT) of the pancreas predominantly Occurs in women, and the occurrence in men is extremely rare. We experienced a male case of SCT. A 38-year-old man was admitted with the complaint of upper abdominal pain. CT scan showed the presence of a mass in the head of the pancreas. The mass was composed of high density areas and low density areas. Ultrasonograms revealed the mass being composed of high echoic areas and low echoic areas. The mass .was hypovascular on angiography. SCT was suspected and pancreaticoduodenectomy was performed. The cut surface of the tumor showed mainly cystic degenerative areas containing dark red hemorrhagic materials. Microscopically, there were solid areas in the periphery and pseudopapillary areas in the center. No metastasis was found in the removed lymph nodes. The tumor cells were not stained by Grimelius' silver stain. The tumor cells were positive for alpha-l-antitrypsin (AAT) and neuron-specific enolase (NSE). Pancreatic hormones such as insulin, glucagon, and somatostatin were all negative. Electron micrograph showed that tumor cells were rich in mitochondria. Zymogen granules and neurosecretory granules were not detected. Estrogen receptor (ER) and progesterone receptor (PR) were both negative.


*HPB Surgery*, 1997, Vol. 10, pp. 315–321
Reprints available directly from the publisher
Photocopying permitted by license only

© 1997 OPA (Overseas Publishers Association)
Amsterdam B.V. Published in The Netherlands
by Harwood Academic Publishers
Printed in India

***Case Report***

### Solid and Cystic Tumor (SCT) of the Pancreas in an Adult Man

K. OHIWA[a], M. IGARASHI[a], N. NAGASUE[b], M. NAGASAKI[c] and T. HARADA[d]

[a]*Department of Surgery, Muikaichi Hospital, Muikaichi, Japan;* [b]*Department of Surgery 2nd Unit, Department of Pathology,* [c]*1st and* [d]*2nd Units, Shimane Medical University, Izumo, Japan*

*(Received 7 July 1996)*

**Solid and cystic tumor (SCT) of the pancreas predominantly occurs in women, and the occurrence in men is extremely rare. We experienced a male case of SCT. A 38-year-old man was admitted with the complaint of upper abdominal pain. CT scan showed the presence of a mass in the head of the pancreas. The mass was composed of high density areas and low density areas. Ultrasonograms revealed the mass being composed of high echoic areas and low echoic areas. The mass was hypovascular on angiography. SCT was suspected and pancreaticoduodenectomy was performed. The cut surface of the tumor showed mainly cystic degenerative areas containing dark red hemorrhagic materials. Microscopically, there were solid areas in the periphery and pseudopapillary areas in the center. No metastasis was found in the removed lymph nodes. The tumor cells were not stained by Grimelius' silver stain. The tumor cells were positive for alpha-1-antitrypsin (AAT) and neuron-specific enolase (NSE). Pancreatic hormones such as insulin, glucagon, and somatostatin were all negative. Electron micrograph showed that tumor cells were rich in mitochondria. Zymogen granules and neurosecretory granules were not detected. Estrogen receptor (ER) and progesterone receptor (PR) were both negative.**

*Keywords:* Solid and cystic tumor, pancreas, immunohistochemistry, electron microscopy, sex hormone receptor

### INTRODUCTION

Solid and cystic tumor (SCT) of the pancreas is a rare tumor characterized by its predominance in young women and a favorable prognosis. We experienced a male case of SCT of the pancreas. In this paper we reported the clinicopathological findings and sex hormone receptor contents in this tumor.

### CASE REPORT

On July 20, 1993, a 38-year-old man was admitted to Muikaichi Hospital with the complaint of upper abdominal pain. On physical

Correspondence: Kanji Ohiwa, MD, Department of Surgery, Muikaichi Hospital, 368-4 Muikaichi-cho, Kanoashi-gun, Shimane 699-55, Japan.

examination, a mass with tenderness was palpated in the right upper quadrant. The mass was 5 cm in diameter, smoothly surfaced, elastic soft, and well circumscribed. Results of laboratory studies were normal except for a white blood cell 13800/mm$^2$ (normal value 4000 to 8000), serum amylase 104IU/l (normal value 27 to 75), serum trypsin 668 ng/ml (normal value 100 to 500), and pancreatic function diagnoatant (PFD) test 51.9% (normal value more than 73.4). Computed tomography (CT) scan showed the presence of a 5 × 5 cm well circumscribed mass in the head of the pancreas. The mass was composed of high density areas and low density areas with a partial rim of calcification. The pancreatic and bile duct were not dilated (Fig. 1). Ultrasonograms revealed a mass being composed of high echoic areas and low echoic areas in the pancreatic head (Fig. 2). Arteriography of the celiac artery demonstrated it as a hypovascular mass with stretched arterial branches of the pancreatic head arcade (Fig. 3). SCT of the pancreas was suspected and a laparotomy was performed on July 30, 1993. An encapsulated tumor was found in the head of the pancreas. There was no evidence of metastases to the liver and lymph nodes macroscopically. Pancreaticoduodenectomy was performed with gross lymph node dissection. Postoperative course was uneventful, and the patient is alive without apparent recurrence two year and 11 months after operation.

Macroscopically, the tumor measured 5 ×5.5 cm. The cut surface of the tumor showed mainly cystic degenerative areas containing dark red hemorrhagic materials. The tumor was surrounded by a fibrous capsule, as thick as 4 mm (Fig. 4). Microscopically, there were solid areas in the periphery and pseudopapillary areas in the center. The pseudopapillary areas consisted of fibrovascular stalks with centrally located capillaries and epithelial covering of several layers of neoplastic cells (Fig. 5a). The solid areas were composed of sheets of neoplastic cells with focal pseudorosette structures surrounding the

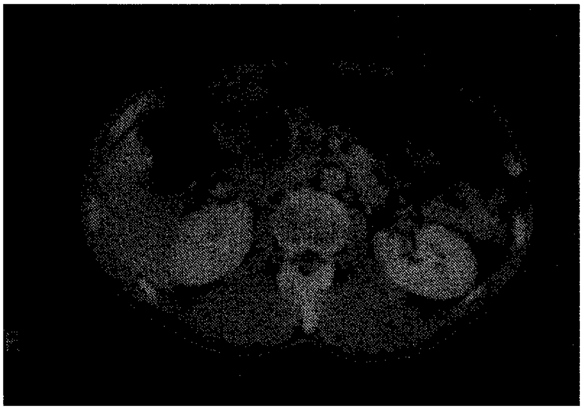

FIGURE 1 CT scan showed the presence of a mass in the head of the pancreas. The mass was composed of high density areas and low density areas with a partial rim of calcification.

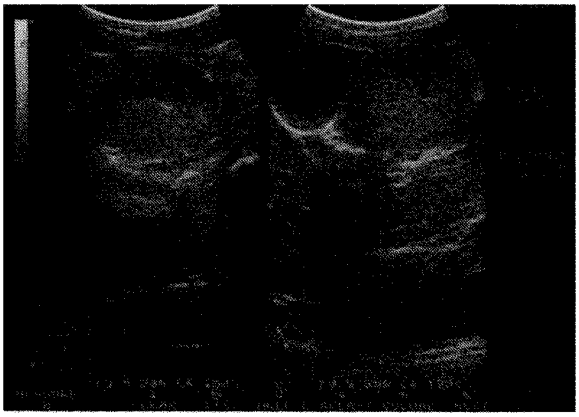

FIGURE 2 Ultrasonography revealed a mass being composed of high echoic areas and low echoic areas in the pancreatic head.

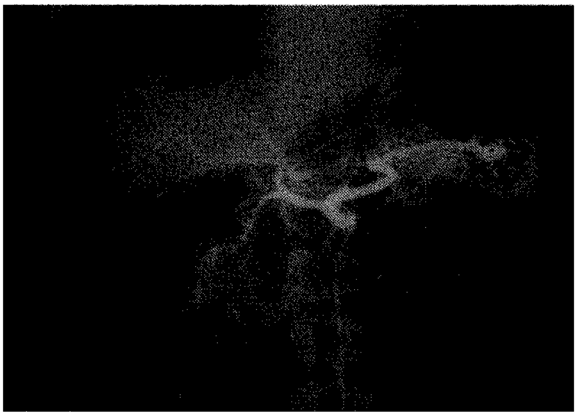

FIGURE 3 Arteriography of the celiac artery demonstrated the tumor as a hypovascular mass with stretched arterial branches of the pancreatic head arcade.

capillary (Fig. 5b). The fibrous capsule was not invaded by the neoplasm. No metastasis was found in the removed lymph nodes microscopically. The tumor cells were not stained by Grimelius' silver stain. The histological diagnosis was SCT of the pancreas.

Immunohistochemical studies were performed on formalin-fixed paraffin-embedded tissue by means of either the avidin-biotin-peroxidase complex (ABC) technique or the peroxidase-antiperoxidase (PAP) technique. Antibodies used in this study were as follows: rabbit antihuman antibodies for neuron-specific enolase (NSE) (Nichirei Co., Ltd., Tokyo, Japan), rabbit antihuman antibodies for the pancreatic hormones such as insulin, glucagon, and somatostatin (Dako Corp., Santa Barbara, CA), biotin-goat anti rabbit IgG (Dakopatts, Copenhagen, Denmark), peroxidase-conjugated streptoavidin (Dakopatts), goat antihuman antibodies for alpha-1-antitrypsin (AAT) (TAGO Co., Inc., Burlingame, CA), rabbit anti-goat serum protein (Dako Corp.), horseradish peroxidase (HRP)-goat anti-HRP complex (Dako Corp.). Control staining was performed by replacement of the first antibody with nonimmune rabbit or goat sera. Sections of a normal pancreas were used as positive controls for the antisera against pancreatic hormones. Most of the tumor cells were diffusely positive for AAT (Fig. 6a) and NSE (Fig. 6b). Pancreatic hormones such as insulin, glucagon, and somatostatin were all negative.

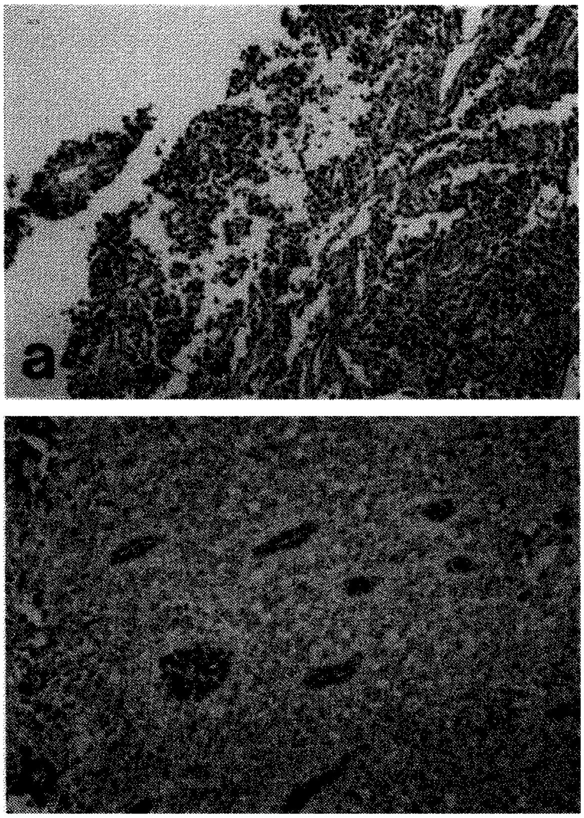

FIGURE 5 Light microscopic features. (a) The pseudopapillary areas consisted of fibrovascular stalks with centrally located capillaries and epithelial covering of several layers of neoplastic cells. HE, × 109 (b) The solid areas were composed of sheets of neoplastic cells with focal pseudorosette structures surrounding the capillary. HE, × 165.

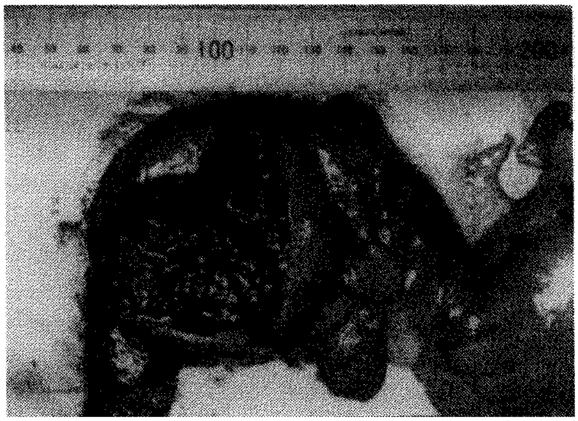

FIGURE 4 The cut surface of the tumor showed mainly cystic degenerative areas containing dark red hemorrhagic materials. The tumor was surrounded by a fibrous capsule.

Electron microscopy was performed on formalin-fixed material which was post-fixed in 1% osmium tetroxide, embedded in Epon-812, and stained with uranyl acetate and lead citrate. Electron micrograph showed that tumor cells were rich in mitochondria. Zymogen granules and neurosecretory granules were not detected (Fig. 7).

Specimens for hormone-receptor analysis were obtained during surgery from the tumor mass. Samples were immediately frozen in liquid nitrogen and kept at −80°C until sent to

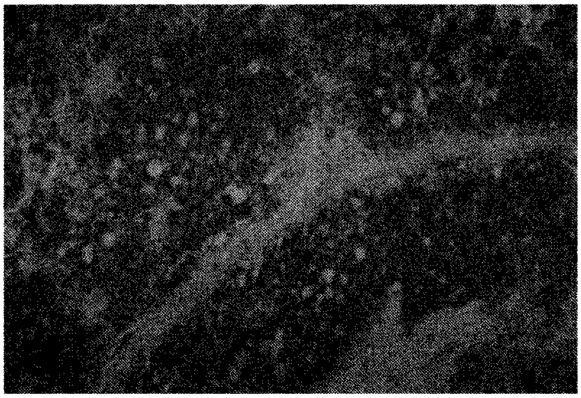

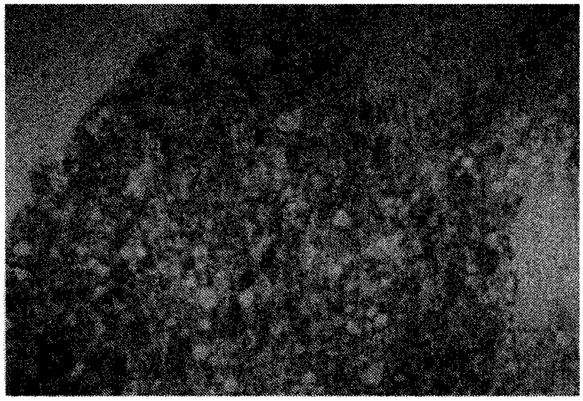

FIGURE 6 Immunohistochemical features. Most of the tumor cells were diffusely positive for alpha-1-antitrypsin (AAT) (a) and neuron-specific enolase (NSE) (b). × 218.

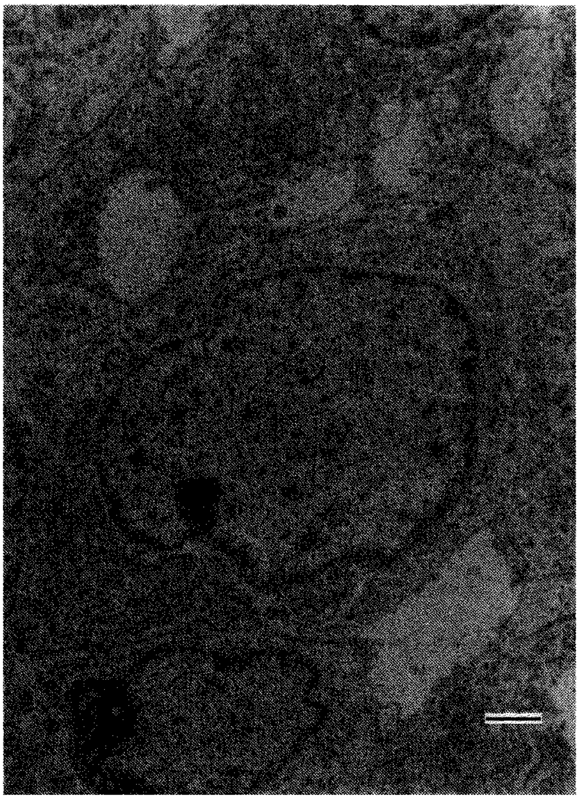

FIGURE 7 Electron micrograph showed that tumor cells were rich in mitochondria. Zymogen granules and neurosecretory granules were not detected. Bar=1 μm.

the Iwakuni Medical Laboratory (Yamaguchi, Japan). Estrogen receptor (ER) and progesterone receptor (PR) were assayed by the enzyme immunoassay (EIA) using monoclonal antibodies (Dainabot Co., Ltd., Tokyo, Japan). ER and PR contents in the tumor were less than 5.0 fmol/mg protein (normal value < 13) and less than 5.0 fmol/mg protein (normal value < 10), respectively.

### DISCUSSION

Frantz [1] initially reported the pancreatic tumor that occurred predominantly in young women with a favorable prognosis as "papillary tumor of the pancreas" in 1959. Since then, several synonyms have appeared for this tumor [2–11]. The name of "solid and cystic tumor (SCT)" called by Klöppel and Morohoshi [12] is widely accepted in our country (Tab. I). Klöppel *et al.*[6] observed positive reaction for alpha-1-antitrypsin (AAT) by immunohistochemistry and zymogen granules by electron microscopy, and they thought that the tumor was of acinar cell origin, and at first named it as "solid and cystic acinar cell tumor" in 1981. Neurosecretory granules were observed in the cytoplasm of this tumor by Schlosnagle *et al.* [7]. However, some investigators reported that there were neither zymogen nor neurosecretory granules in the cytoplasm of this tumor [2, 4, 5, 8, 11]. Neuron-specific enolase (NSE) was found in the tumor by Chott *et al.* [13]. Morohoshi *et al.* [12] emphasized that AAT did not serve as a specific marker for acinar cell tumors, and later corrected the name to "solid and cystic tumor (SCT)" in 1984. The histogenesis of this tumor has not been completely clarified, but it is suspected that this tumor is derived from primordial cells that can differentiate to acinar cell, ductal cells, or endocrine cells [11]. Anyhow, the clinicopathological findings of our male case agree with previously reported findings of SCT in women.

SCT predominantly occurs in women, and the occurrence in men is extremely rare. In Japanese literature that included 126 cases, only 10 patients (7.9%) were males [14]. Because of this predominant occurrence of SCT in women, some investigators thought that hormonal factors might play a role in its pathogenesis (Tab. II). Ladanyi *et al.* [15] found a definite increase in both ER and PR in this tumor. Wrba *et al.* [16] demonstrated negativity for ER but strong positivity for PR. Carbone *et al.* [17] found high levels of type II ER, while type I ER were absent or present at very low levels. However, ER was not detected by Miettinen *et al.* [18]. Neither ER nor PR were found by Katz *et al.* [19], and Pettinato *et al.* [20]. Klöppel *et al.* [21] examined

TABLE I Synonyms of the solid and cystic tumor (SCT)

| | |
|---|---|
| papillary tumor | Frantz (1959)[1] |
| papillary epithelial neoplasm | Hamoudi (1970)[2] |
| adenocarcinoma in childhood | Taxy (1976)[3] |
| papillary-cystic neoplasm | Boor (1979)[4] |
| solid and papillary epithelial neoplasm | Compagno (1979) [5] |
| solid and cystic acinar cell tumor | Klöppel (1981) [6] |
| papillary and solid neoplasm | Schlosnagle (1981) [7] |
| papillary-cystic epithelial neoplasm | Alm (1981)[8] |
| papillary-cystic carcinoma | Dales (1983)[9] |
| solid and papillary neoplasm | Sanfey (1983)[10] |
| papillary cystic tumor | Morrison (1984)[11] |

TABLE II Reports of sex hormone receptors in (SCT)

| Study | Age/Sex | Methods | Estrogen receptor | Progesterone receptor |
|---|---|---|---|---|
| Ladanyi [15] | 18/F | Dextran-coated charcoal method | 19.2 fmol/mg protein | 268.3 fmol/mg protein |
| Wrba [16] | 16/F | Dextran-coated charcoal method | 0 fmol/mg protein | 112 fmol/mg protein |
| Carbone [17] | 17/F | Dextran-coated charcoal method | typeII: 512 fmol/mg protein | 18 fmol/mg protein |
| | | Immunohistochemical method | Negative | |
| | 28/F | Dextran-coated charcoal method | TypeI: 5.1 fmol/mg protein<br>TypeII: 795 fmol/mg protein | 61 fmol/mg protein |
| | | Immunohistochemical method | Negative | |
| Miettinen [18] | 20/F | Immunohistochemical method | Negative | |
| | 34/F | Immunohistochemical method | Negative | |
| Katz [19] | 26/F | Dextran-coated charcoal method | Less than 3 fmol/mg protein | Less than 3 fmol/mg protein |
| Pettinato [20] | (15 cases) | Immunohistochemical method | Negative | Negative |
| Klöppel [21] | 25/M | Immunohistochemical method | Negative | Negative |
| | 25/M | Immunohistochemical method | Negative | Negative |
| Present study | 38/M | Enzyme immunoassay | Less than 5.0 fmol/mg protein | Less than 5.0 fmol/mg protein |

ER and PR in SCT of male patients using paraffin-embedded specimens, and did not detect ER and PR. There exists little literature on examining ER and PR using fresh frozen specimens in SCT of male patients. In this study, we assayed ER and PR in SCT of a male patient by EIA using fresh frozen specimens, and demonstrated that both ER and PR were negative. Our results support the results by Klöppel *et al*. Estrogen and progesterone may not play a role in the pathogenesis of SCT.

The treatment of SCT is complete resection of the tumor. Panceaticoduodenectomy for lesions of the head of the pancreas and distal pancreatectomy for lesions of the body and tail of the pancreas should be performed. The prognosis after complete resection is favorable. However, this tumor should be considered as a low-grade malignancy because liver metastasis, lymph node metastasis, and peritonitis carcinomatosa have been reported [22–25].

### *References*

[1] Frantz, V. K. (1959). Tumor of the pancreas. In Atlas of Tumor Pathology VII, edited by Blumberg, C. W. fasc. 27, pp.32–33. Washington, DC: Armed Forces Institute of Pathology.

[2] Hamoudi, A. B., Misugi, K., Grosfeld, J. L. and Reiner, C. B. (1970). Papillary epithelial neoplasm of pancreas in a child. Report of a case with electron microscopy, *Cancer*, **26**, 1126–1134.

[3] Taxy, J. B. (1976). Adenocarcinoma of the pancreas in childhood. Report of a case and a review of the English language literature, *Cancer*, **37**, 1508–1518.

[4] Boor, P. J. and Swanson, M. R. (1979). Papillary-cystic neoplasm of the pancreas, *Am. J. Surg. Pathol.*, **3**, 69–75.

[5] Compagno, J., Oertel, J. E. and Kremzar, M. (1979). Solid and papillary epithelial neoplasm of the pancreas, probably of the small duct origin: A clinicopathologic study of 52 cases, *Lab Invest*, **40**, 248–249.

[6] Klöppel, G., Morohoshi, T., John, H. D., Oehmichen, W., Opitz, K., Angelkort, A., Lietz, H. and Rückert, K. (1981). Solid and cystic acinar cell tumour of the pancreas, A tumour in young woman with favourable prognosis. *Virchow Arch. [A]*, **392**, 171–183.

[7] Schlosnagle, D. C. and Campbell, W. G. (1981). The papillary and solid neoplasm of the pancreas: A report of two cases with electron microscopy, one containing neurosecretory granules, *Cancer*, **47**, 2603–2610.

[8] Alm, P., Jönsson, P.-E, Karp, W., Lindberg, L.-G., Stenram, U. and Sundler, F. (1981). A case of papillary-cystic epithelial neoplasm of the pancreas, *Act. Pathol. Microbiol. Scand. [A]*, **89**, 125–132.

[9] Dales, R. L., Garcia, J. C. and Davies, R. S. (1983). Papillary-cystic carcinoma of the pancreas, *J. Surg. Oncol.*, **22**, 115–117.

[10] Sanfey, H., Mendelsohn, G. and Cameron, J. L. (1983). Solid and papillary neoplasm of the pancreas. A potentially curable surgical lesion, *Ann. Surg.*, **197**, 272–275.

[11] Morrison, D. M., Jewell, L. D., McCaughey, W. T. E., Danyluk, J., Shnitka, T. K. and Manickavel, V. (1984). Papillary cystic tumor of the pancreas, *Arch. Pathol. Lab. Med.*, **108**, 723–727.

[12] Morohoshi, T., Kanda, M., Horie, A., Chott, A., Dreyer, T., Klöppel, G. and Heitz, P. U. (1987). Immunocytochemical markers of uncommon pancreatic tumors. Acinar cell carcinoma, pancreatoblastoma, and solid cystic (papillary-cystic) tumor, *Cancer*, **59**, 739–747.

[13] Chott, A., Klöppel, G., Buxbaum, P. and Heitz, P.U. (1987). Neuron specific enolase demonstration in the diagnosis of a solid-cystic (papillary cystic) tumour of the pancreas, *Virchows Arch [A]*, **410**, 397–402.

[14] Sutoh, K., Ouchi, A., Tokumura, H., Imaoka, Y., Yamamoto, K. and Matsushiro, T. (1994). Solid and cystic tumor of the pancreas in a male, *Jpn. J. Gastroenterol Surg.*, **27**, 1848–1852 (in Japanese).

[15] Ladanyi, M., Mulay, S., Arseneau, J. and Bettez, P. (1987). Estrogen and progesterone receptor determination in the papillary cystic neoplasm of the pancreas with immunohistochemical and ultrastructural observations, *Cancer*, **60**, 1604–1611.

[16] Wrba, F., Chott, A., Ludvik, B., Schratter, M., Spona, J., Reiner, A., Schernthaner, G. and Krisch, K. (1988). Solid and cystic tumour of the pancreas; a hormonal-dependent neoplasm? *Histopathology*, **12**, 338–340.

[17] Carbone, A., Ranelletti, F. O., Rinelli, A., Vecchio, F. M., Lauriola, L., Piantelli, M. and Capelli, A. (1989). Type II estrogen receptors in the papillary cystic tumor of the pancreas, *Am. J. Clin. Pathol.*, **92**, 572–576.

[18] Miettinen, M., Partanen, S., Fräki, O. and Kivilaakso, E. (1987). Papillary cystic tumor of the pancreas. An analysis of cellular differentiation by electron microscopy and immunohistochemistry, *Am. J. Surg. Pathol.*, **11**, 855–865.

[19] Katz, L. B. K. and Ehya, H. (1990). Aspiration cytology of papillary cystic neoplasm of the pancreas, *Am. J. Clin. Pathol.*, **94**, 328–333.

[20] Pettinato, G., Manivel, J. C., Ravetto, C., Terracciano, L. M., Gould, E. W., Tuoro, A. D., Jaszcz, W. and Albores-Saavedra, J. (1992). Papillary cystic tumor of the pancreas. A clinicopathological study of 20 cases with cytologic, immunohistochemical, ultrastructural, and flow cytometric observations, and a review of the literature, *Am. J. Clin. Pathol.*, **98**, 478–488.

[21] Klöppel, G., Maurer, R., Hofmann, E., Lüthold, K., Oscarson, J., Forsby, N., Ihse, I., Ljungberg, O. and Heitz, P. U. (1991). Solid-cystic (papillary-cystic) tumours within and outside the pancreas in men: report of two patients, *Virchows Arch. [A]*, **418**, 179–183.

[22] Rustin, R. B., Broughan, T. A., Hermann, R. E., Grundfest-Broniatowski, S. F., Petras, R. E. and Hart, W. R. (1986). Papillary cystic epithelial neoplasms of the pancreas. A clinical study of four cases, *Arch. Surg.*, **121**, 1073–1076.

[23] Matsuda, Y., Imai, Y., Kawata, S., Nishikawa, M., Miyoshi, S., Saito, R., Minami, Y. and Tarui, S. (1987). Papillary-cystic neoplasm of the pancreas with multiple hepatic metastases: A case report, *Gastroenterol. Jpn.*, **22**, 379–384.

[24] Matsunou, H. and Konishi, F. (1990). Papillary-cystic neoplasm of the pancreas. A clinicopathologic study concerning the tumor aging and malignancy of nine cases, *Cancer*, **65**, 283–291.

[25] Stömmer, P., Kraus, J., Stolte, M. and Giedl, J. (1991). Solid and cystic pancreatic tumors. Clinical, histochemical, and electron microscopic features in ten cases, *Cancer*, **67**, 1635–1641.

